# Supplementary material for: Structure of the catalytically active APOBEC3G bound to a DNA oligonucleotide inhibitor reveals tetrahedral geometry of the transition state
Source: Nat Commun. 2022 Nov 19;13:7117. doi: 10.1038/s41467-022-34752-1 (PMC9675756; doi:10.1038/s41467-022-34752-1)
Supplement: Supplementary file 1 — Supplementary Information [file 41467_2022_34752_MOESM1_ESM.pdf]

Supplementary Data:

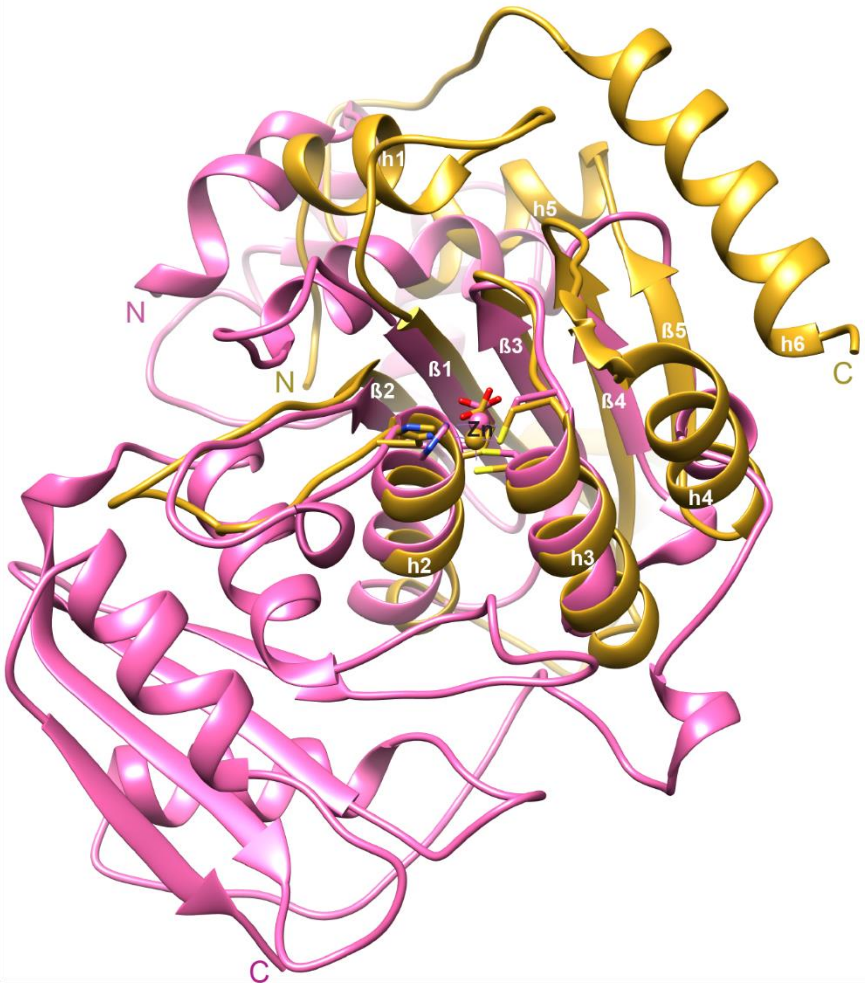

Supplementary Figure 1a. Structure alignment of wild-type human A3G-CTD (PDB ID: 4ROV) with *E. coli* CDA (PDB ID: 1CTU) using the program UCSF Chimera. Protein structures are represented as cartoon (A3G-CTD2 is gold and *E. coli* CDA is pink). Zn^{2+} is shown as a sphere (Orange in A3G-CTD2 and pink in *E. coli* CDA). Zn^{2+} coordinating residues (H257, C288, C291 in A3G-CTD and H102, C129, C132 in *E. coli* CDA) and catalytic residue (E259 in A3G-CTD and E104 in *E. coli* CDA) are shown as sticks. N and C (colored as corresponding protein's color) indicate the N- and C-terminal ends of the protein. Only A3G-CTD secondary structures are labeled. Zn^{2+}, Helix 2, Helix 3, ß1, ß2, ß3 and ß4 of A3G-CTD are structurally aligned with *E. coli* CDA.

```
                                                                   ß1
A3G-CTD    187 GSHMASLRHSM--------D-PPT-------------------------- 201
E.Coli-CDA   1 -----------MHPRFQTAFAQ--LADNLQSALEPILADKYFPALLTGEQ 37

                                                                   ß1
A3G-CTD    202 --------------------FTFNFNNEPWVRGR------------HETY 219
E.Coli-CDA  38 VSSLKSATGLDEDALAFALLP-------------LAAACARTPLSNFNVG 74

                   ß1                    ß2                      h2
A3G-CTD    220 LCYEVERMHNDTWVL-LNQRRGFLCNQAPHKHGF--L---EGRHAELCFL 263
E.Coli-CDA  75 AIARGV---------SG--TWYFGANMEF-----IGATMQQTVHAEQSAI 108

                 h2              ß3          h3
A3G-CTD    264 DVIP-FWKLDLDQDYR--VTCFTSWSPCFSCAQEMAKFISKNKHVS---L 307
E.Coli-CDA 109 SHAWLSGEK-------ALAAITVNYTPCGHCRQFMNEL---N--S-GLDL 145

                  ß4
A3G-CTD    308 CIFTARIYDDQGRCQEG---------LR--TLAEAGAKISIMTYSEFKHC 346
E.Coli-CDA 146 RIHLP------------GREAHALRDYLPDA------------------- 164

A3G-CTD    347 WDTFVDHQGCPFQPWDGLDEHSQDLSGRLRAILQNQEN------------ 384
E.Coli-CDA 165 --------------------------------------FGPKDLEIKTLL 176

A3G-CTD    384 -------------------------------------------------- 384
E.Coli-CDA 177 MDEQDHGYALTGDALSQAAIAAANRSHMPYSKSPSGVALECKDGRIFSGS 226

A3G-CTD    384 -------------------------------------------------- 384
E.Coli-CDA 227 YAENAAFNPTLPPLQGALILLNLKGYDYPDIQRAVLAEKADAPLIQWDAT 276

A3G-CTD    384 ------------------ 384
E.Coli-CDA 277 SATLKALGCHSIDRVLLA 294
```

Supplementary Figure 1b. Structure based sequence alignment of A3G-CTD (PDB ID: 4ROV) with *E. coli* CDA (PDB ID: 1CTU) created by the program UCSF Chimera. Superimposed structures are shown in **Supplemental Fig. 1a**. Structurally aligned sequences (Helix 2, Helix 3, ß1, ß2, ß3 and ß4 of A3G-CTD) are highlighted by yellow. Zn^{2+} coordinating residues (H257, C288, C291 in A3G-CTD and H102, C129, C132 in *E. coli* CDA) and catalytic residue (E259 in A3G-CTD and E104 in *E. coli* CDA) are highlighted by green.

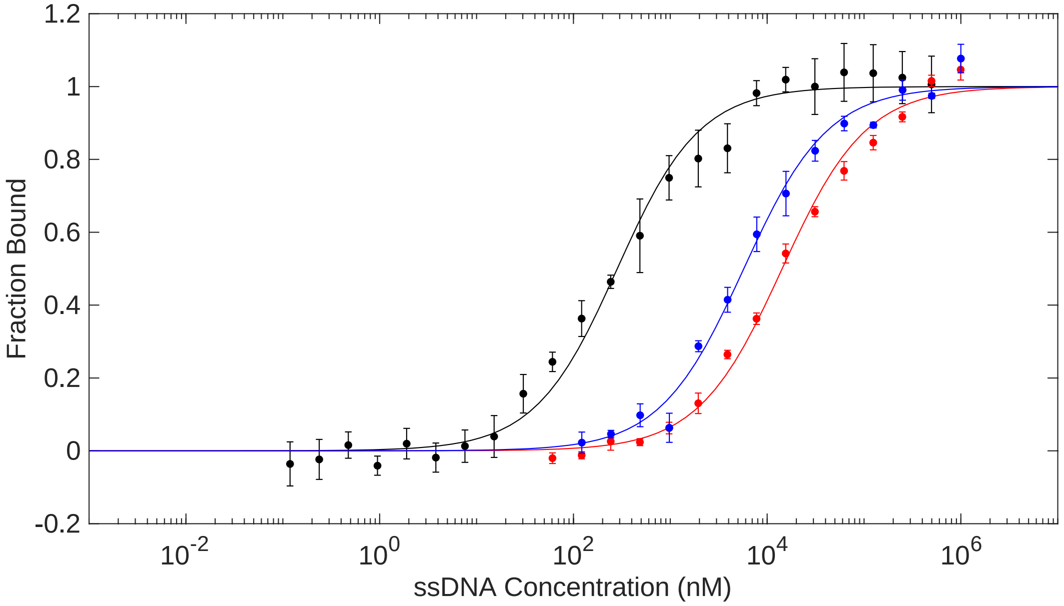

Supplementary Figure 2a. Micro Scale Thermophoresis binding data for 5′-AATCCdZAAA binding to active A3G-CTD2 (black), inactive A3G-CTD2* (red), and 5′-AATCCCAAA substrate binding to inactive A3G-CTD2* (blue). Data are presented as mean values +/- SD. Source data are provided as a Source Data file. n=3 biologically independent samples.

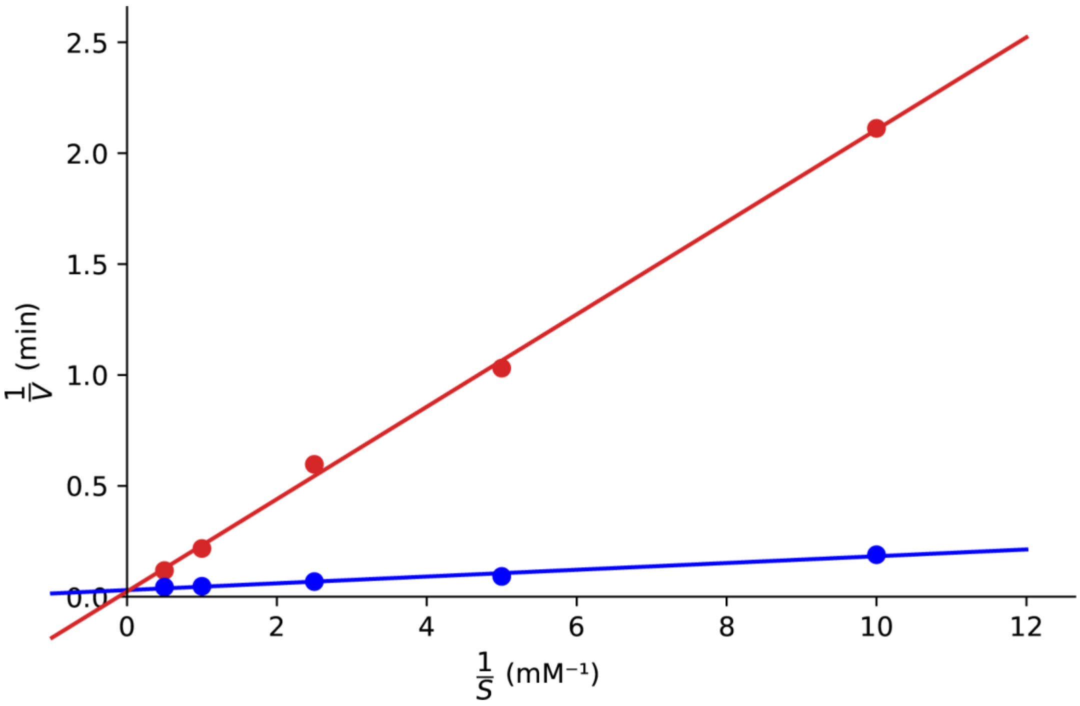

Supplementary Figure 2b. Lineweaver Burk plots of 200 nM A3G-CTD2 in the absence (blue) and presence (red) of 50 µM 5′-AATCCdZAAA confirm competitive inhibition of A3G-CTD2 deaminase activity. Initial deamination rates were measured at 100 µM, 200 µM, 400 µM, 1 mM, and 2 mM 5′-AATCCCAAA substrate concentrations. Source data are provided as a Source Data file. n=3 biologically independent samples.

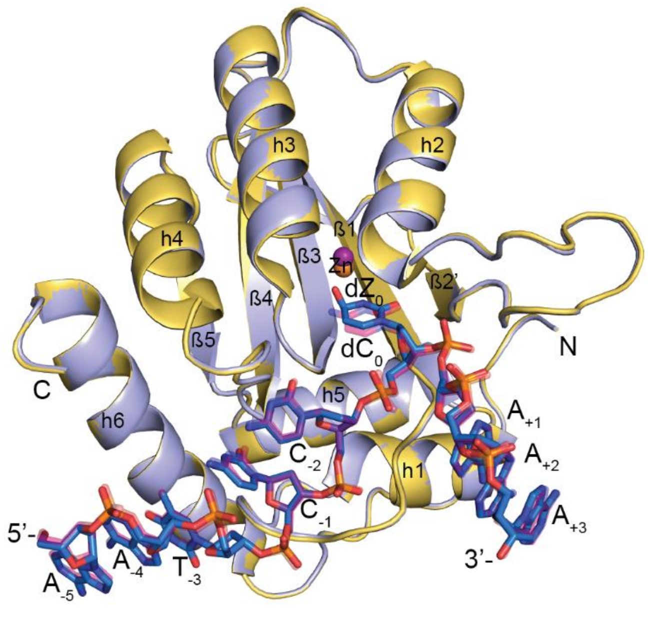

Supplementary Figure 3. Superposition of A3G-CTD2:dZ-ssDNA (PDB ID: 7UXD) structure with A3G-CTD2*:ssDNA (PDB ID: 6BUX) structure. Protein structure represented as cartoon (A3G-CTD2 is yellow and A3G-CTD2* is light blue) and ssDNA structure represented as sticks (dZ-ssDNA as blue and dC-ssDNA as transparent pink). Zn^{2+} shown as a sphere (Orange in A3G-CTD2 and purple in A3G-CTD2*). N and C indicate the N- and C-terminal ends of the protein, 5′- and 3′- indicates 5′ and 3′ ends of the ssDNA.

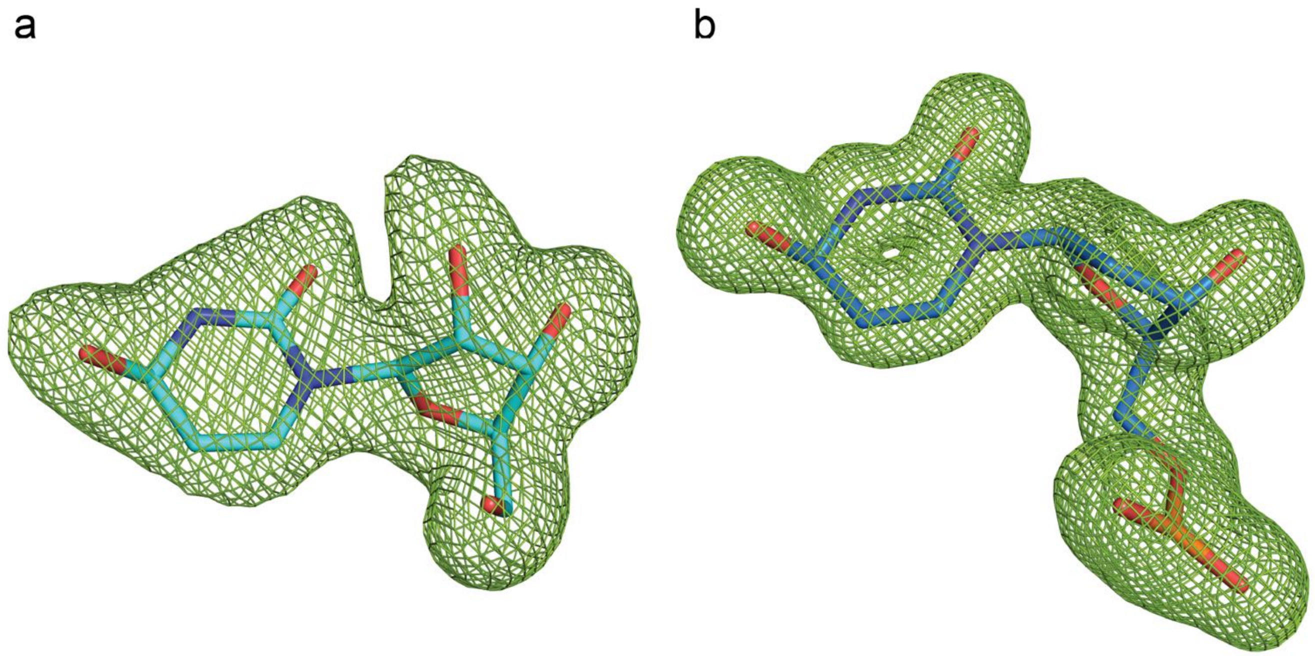

Supplementary Figure 4. Omit map for ligands. a) Omit (Fo–Fc) map contoured at 3σ is shown in green around the ribo-zebularine hydrated intermediate (ZEB-OH) (PDB ID: 1CTU)
b) Omit (Fo–Fc) map contoured at 3σ is shown in green around the 2′-deoxy-zebularine hydrated intermediate (dZ-H_2O) (PDB ID: 7UXD).

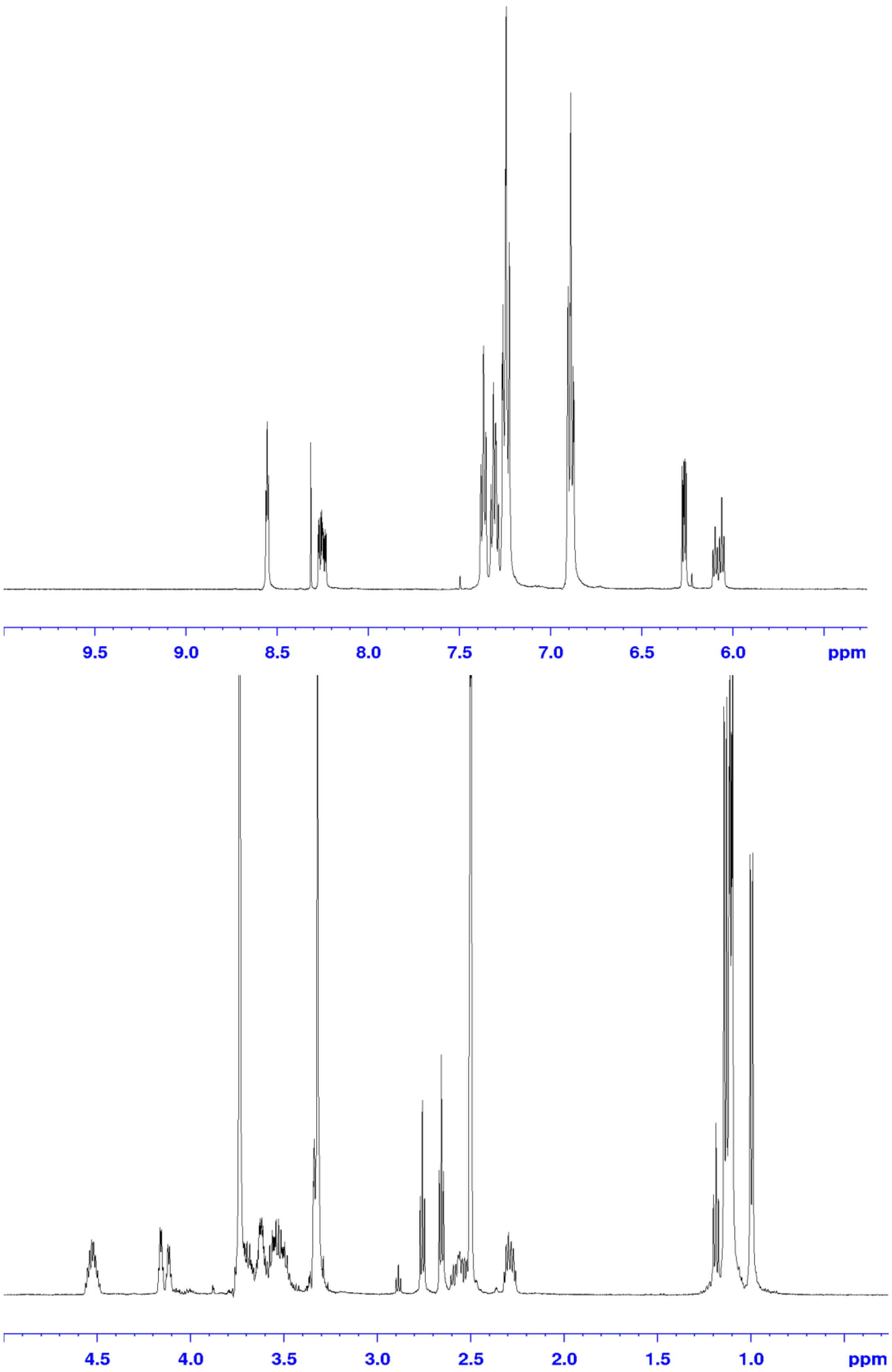

Supplementary Figure 5. ^{1}H NMR spectrum of final dZ phosphoramidite.

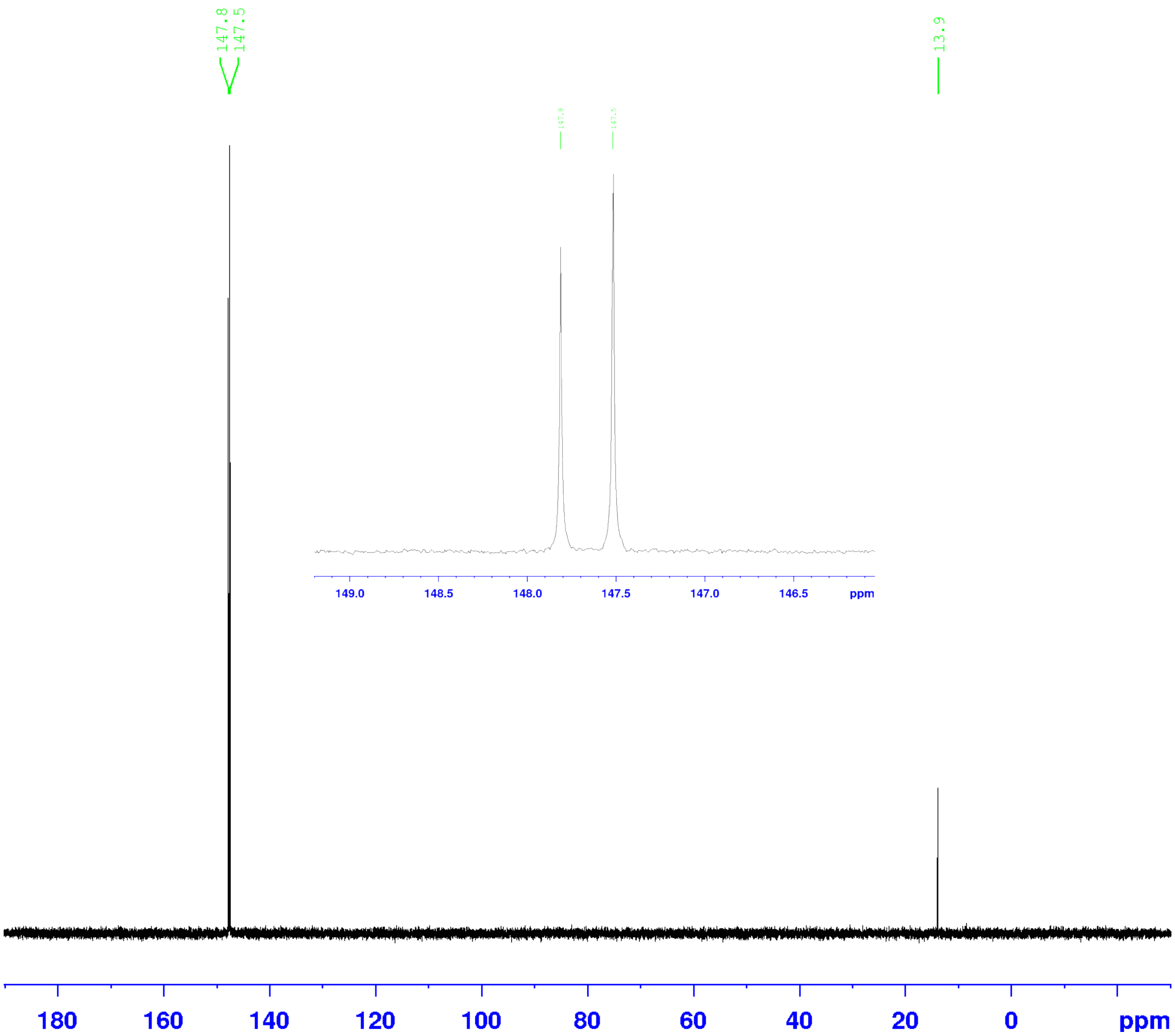

Supplementary Figure 6. ^{31}P NMR spectrum of final dZ phosphoramidite (inset zoom of 146-149ppm).

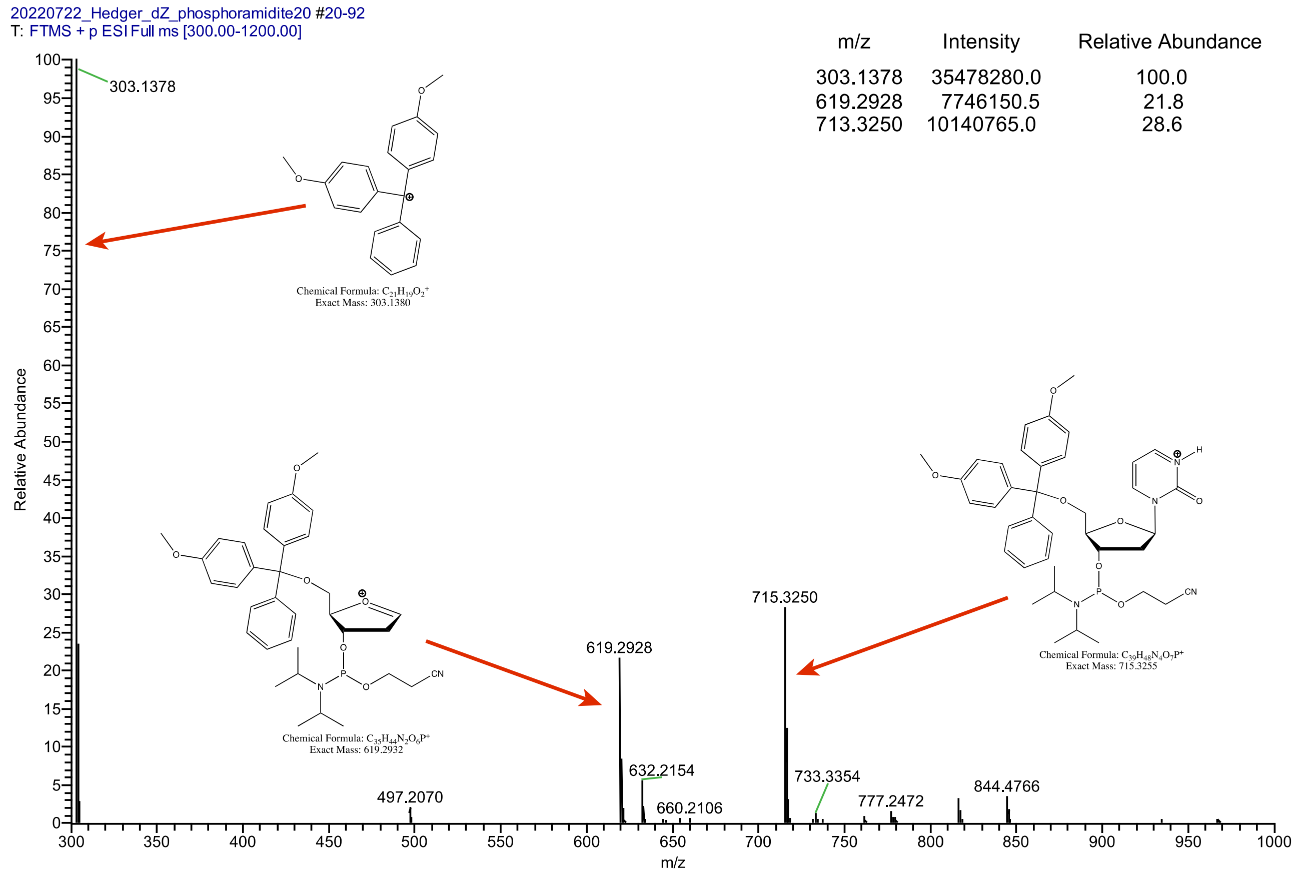

Supplementary Figure 7. HRMS ESI Mass spectrum of final dZ phosphoramidite. Spectrum also shows DMT+ cation and oxocarbenium cation fragments from the main compound.

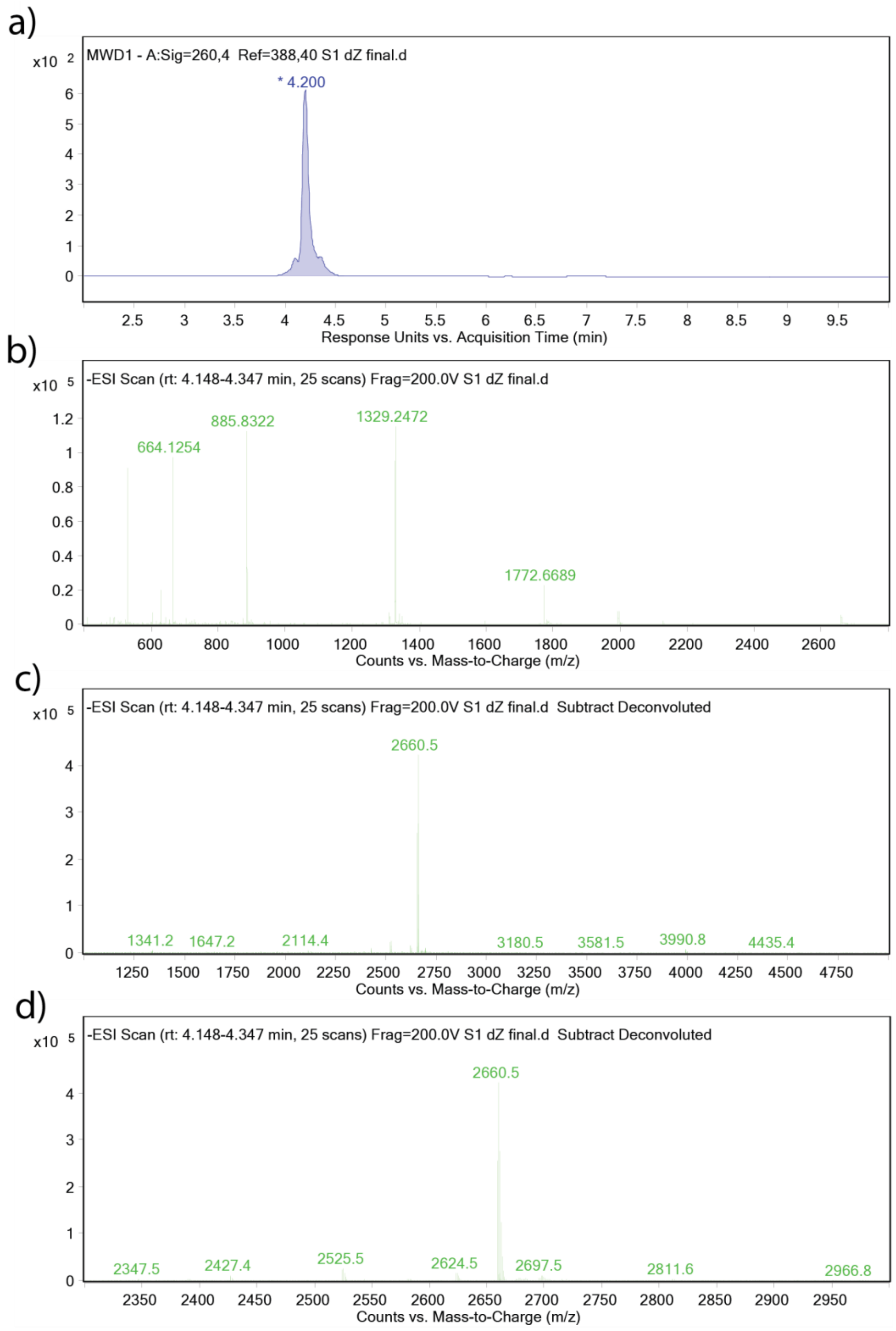

Supplementary Figure 8. Mass spectrometry analysis for dZ containing oligonucleotide (5′-AATCCdZAAA). a) LCMS UV trace at 260 nm. b) Mass spectrum showing multiply charged negative species c) Deconvoluted mass spectrum between 1000-5000 m/z showing the main oligonucleotide peak at 2660.5 d) Same as c, but expanded to focus on the region of 2300-3000 m/z.
